# Impact of Graphene-Based Surfaces on the Basic Biological Properties of Human Umbilical Cord Mesenchymal Stem Cells: Implications for Ex Vivo Cell Expansion Aimed at Tissue Repair

**DOI:** 10.3390/ijms20184561

**Published:** 2019-09-14

**Authors:** Joanna Jagiełło, Małgorzata Sekuła-Stryjewska, Sylwia Noga, Edyta Adamczyk, Monika Dźwigońska, Magdalena Kurcz, Katarzyna Kurp, Magdalena Winkowska-Struzik, Elżbieta Karnas, Dariusz Boruczkowski, Zbigniew Madeja, Ludwika Lipińska, Ewa K. Zuba-Surma

**Affiliations:** 1 Department of Chemical Synthesis and Flake Graphene, Łukasiewicz Research Network - Institute of Electronic Materials Technology, 01-919 Warsaw, Poland; joanna.jagiello@itme.edu.pl (J.J.);; 2 Malopolska Centre of Biotechnology, Jagiellonian University, 30-387 Krakow, Poland; malgorzata.sekula@uj.edu.pl (M.S.-S.);; 3 Department of Cell Biology, Faculty of Biochemistry, Biophysics and Biotechnology, Jagiellonian University, 30-387 Krakow, Polandz.madeja@uj.edu.pl (Z.M.); 4 Polish Stem Cell Bank, 00-867 Warsaw, Poland

**Keywords:** graphene oxide, reduced graphene oxide, human umbilical cord mesenchymal stem/stromal cells, tissue engineering, regeneration, imaging

## Abstract

The potential therapeutic applications of mesenchymal stem/stromal cells (MSCs) and biomaterials have attracted a great amount of interest in the field of biomedical engineering. MSCs are multipotent adult stem cells characterized as cells with specific features, e.g., high differentiation potential, low immunogenicity, immunomodulatory properties, and efficient in vitro expansion ability. Human umbilical cord Wharton’s jelly-derived MSCs (hUC-MSCs) are a new, important cell type that may be used for therapeutic purposes, i.e., for autologous and allogeneic transplantations. To improve the therapeutic efficiency of hUC-MSCs, novel biomaterials have been considered for use as scaffolds dedicated to the propagation and differentiation of these cells. Nowadays, some of the most promising materials for tissue engineering include graphene and its derivatives such as graphene oxide (GO) and reduced graphene oxide (rGO). Due to their physicochemical properties, they can be easily modified with biomolecules, which enable their interaction with different types of cells, including MSCs. In this study, we demonstrate the impact of graphene-based substrates (GO, rGO) on the biological properties of hUC-MSCs. The size of the GO flakes and the reduction level of GO have been considered as important factors determining the most favorable surface for hUC-MSCs growth. The obtained results revealed that GO and rGO are suitable scaffolds for hUC-MSCs. hUC-MSCs cultured on: (i) a thin layer of GO and (ii) an rGO surface with a low reduction level demonstrated a viability and proliferation rate comparable to those estimated under standard culture conditions. Interestingly, cell culture on a highly reduced GO substrate resulted in a decreased hUC-MSCs proliferation rate and induced cell apoptosis. Moreover, our analysis demonstrated that hUC-MSCs cultured on all the tested GO and rGO scaffolds showed no alterations of their typical mesenchymal phenotype, regardless of the reduction level and size of the GO flakes. Thus, GO scaffolds and rGO scaffolds with a low reduction level exhibit potential applicability as novel, safe, and biocompatible materials for utilization in regenerative medicine.

## 1. Introduction

Since some of the unusual properties of graphene were examined and described by A. Geim and K. Novoselov in 2004, it has emerged as one of the most widely studied carbon nanomaterials [1,2] Due to its two-dimensional structure with carbon atoms arranged in a hexagonal crystal lattice with sp^2^ hybridization, graphene has been reported to possess a lot of extraordinary properties, e.g., a large theoretical specific surface area (2630 m^2^·g^−1^), high theoretical intrinsic mobility (200,000 cm^2^·v^−1^·s^−1^), high Young′s modulus (∼1.0 TPa), high thermal conductivity (∼5000 Wm^−1^·K^−1^), high optical transmittance (∼97.7%) [3], low electrical resistivity (10^−6^ Ω·cm), and electron mobility values reported in excess of 15,000 cm^2^·v^−1^·s^−1^ at room temperature [4]. Recently, an increasing amount of attention is being paid to graphene derivatives, such as graphene oxide (GO) and reduced graphene oxide (rGO). Due to their altered structure, with functional oxide groups bound to their surface, they possess properties different from those of ideal graphene, thereby making them suitable for several medical applications [5]. Moreover, the ease of their synthesis and their ability to be modified are huge advantages that make GO and rGO more suitable for applications. In our study, GO was produced by methods based on those proposed by Hummers [6] and Marcano [7], which allow the production of nanometer- and micrometer-sized flakes. Oxidation was performed via a chemical route using graphite flakes as the starting material and the inorganic compounds H_2_SO_4_, H_3_PO_4_, and KMnO_4_ as exfoliating and oxidizing chemicals. The characteristic properties of GO include hydrophilicity and the lack of electrical conductivity, which can be restored by a reduction process. There are different techniques for the reduction of GO: the high reduction of the GO can be achieved by using chemicals, e.g., hydrazine or metal hydrides [8], or the low reduction of the GO may be obtained by using biocompatible compounds, e.g., amino acids [9], carrot root [10], and glucose [11].

Mesenchymal stem/stromal cells (MSCs) represent a class of adult multipotent stem cells; they possess the ability to differentiate into different cell types of mesodermal origin, including osteoblasts, chondrocytes, and adipocytes [12,13]. MSCs can be isolated from different sources, such as the bone marrow, peripheral blood, adipose tissue, umbilical cord blood, the Wharton’s jelly within the umbilical cord, and others [14,15,16]. Growing evidence has demonstrated potential therapeutic applications of MSCs derived from the Wharton’s jelly in the human umbilical cord (hUC-MSCs) for cell-based therapies in humans. Interestingly, hUC-MSCs exhibit several advantages over other stem cell tissue sources. In particular, hUC-MSCs represent no ethical concerns, are easy to harvest, and demonstrate low immunogenicity and high proliferative capacity, as well as the ability to differentiate into several specialized cell types in in vitro conditions [17,18,19]. Moreover, hUC-MSCs possess a huge therapeutic potential, leading to their utilization for autologous and allogenic transplantations [20,21].

Due to the physicochemical characteristics of GO and rGO (including amphiphilicity, honeycomb structure, and surface chemistry), graphene-based scaffolds are able to interact with biomolecules and cells [22]. Moreover, the biofunctionalization of graphene surfaces may promote these interactions through the enhancement of cell adhesion and proliferation [23,24]. However, the most significant factors that influence the biocompatibility of graphene derivatives have still not been comprehensively investigated. Therefore, the chemical (e.g., oxidation degree, lattice, and edge defects) and morphological (e.g., flake size, wrinkles, and agglomeration) [25,26,27] aspects are of great interest for the development of a novel graphene-based material that may be used as an artificial niche that modulates the fate and behavior of stem cells. It is well known that the culture surface is crucial in stem cell research, because it may modulate cell differentiation or maintain the self-renewal ability and pluripotency of stem cells [28,29]. However, the interactions of hUC-MSCs with selected GO and rGO surfaces that are currently investigated for tissue repair have not yet been comprehensively studied. Thus, the objective of this study was to determine the most suitable graphene-based scaffold dedicated to the growth and propagation of hUC-MSCs. In this study, the influence of GO and rGO surfaces on several biological properties of hUC-MSCs has been examined in order to improve the therapeutic efficacy of these cells for tissue engineering applications.

## 2. Results

### 2.1. Physicochemical Characteristics of the GO and rGO Samples

#### 2.1.1. SEM Analysis

**Figure 1:**
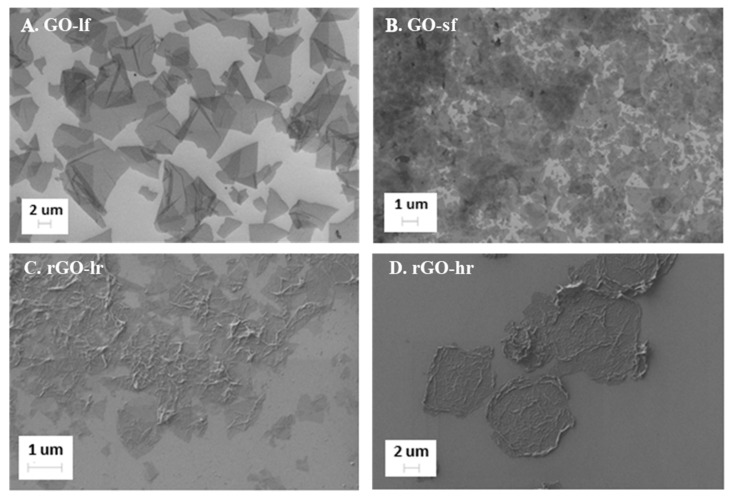
SEM images of the graphene oxide (GO) with large flakes (GO-lf, panel **A**), GO with small flakes (GO-sf, panel **B**), reduced graphene oxide (rGO) with low reduction level (rGO-lr, panel **C**), and rGO with high reduction level (rGO-hr, panel **D**) placed on a Si substrate. Different sizes of the GO flakes (**A,B**) and the aggregation tendency of the rGO flakes, (**C,D**) are shown.

The morphology of the GO and rGO samples was examined by SEM analysis and the obtained results are shown in Figure 1. The GO flakes were distributed uniformly on the Si substrate; this provided information concerning their shape and size, and indirectly, information regarding their thickness. The particles of the GO-sf and GO-lf samples were of different sizes, consistent with our expectations. The size of the large flakes (Figure 1A) ranged from 10–20 µm, and the smaller flakes (Figure 1B) were 0.2–2 µm in size; the difference between the size of these flakes was about 10-fold. Moreover, the images indicate that the GO samples were well dispersed. This is due to the repulsive electrostatic interactions among the negatively polarized oxygen groups covalently bonded to the GO surface. The rGO flakes (Figure 1C,D) were more agglomerated, reflecting their natural tendency after the partial removal of the oxygen groups during GO reduction. A more effective reduction leads to a greater agglomeration of the flakes (Figure 1D).

#### 2.1.2. Raman Spectroscopy

**Figure 2:**
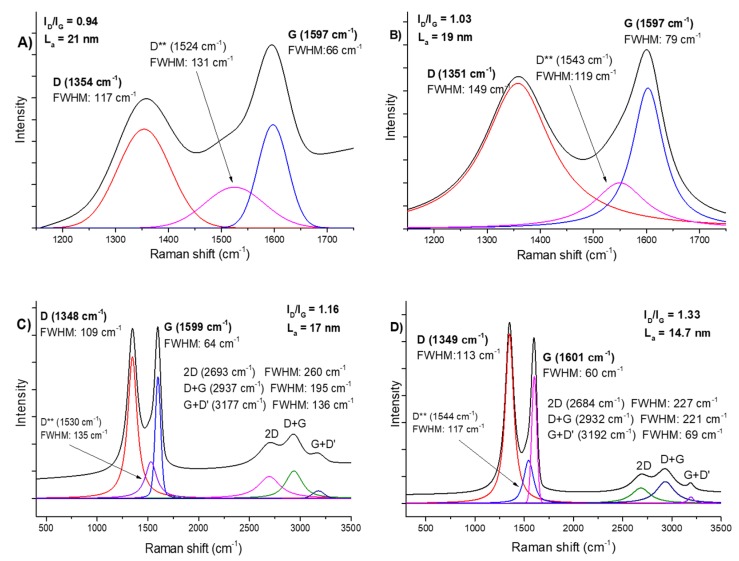
Raman spectra of the GO-sf (**A**), GO-lf (**B**), rGO-lr (**C**), and rGO-hr (**D**) samples. Legend: GO-lf: graphene oxide with large flakes (10–20 µm), GO-sf: graphene oxide with small flakes (0.2–2 µm), rGO-lr: reduced graphene oxide with low reduction level, and rGO-hr: reduced graphene oxide with high reduction level. The black lines in spectra presented in the Figure 2 (A, B, C, D) correspond peak sums, the colored lines depict single mods. For example, in Figure 2A: the red line corresponds to peak D, the pink line peak D **, the blue line peak G.

Raman spectroscopy was used to estimate the degree of GO reduction in the rGO-lr and rGO-hr samples and to find structural differences between the GO-lf and GO-sf samples (Figure 2). The main bands for the GO and rGO samples appeared at about 1350 cm^−1^ (D band) and about 1600 cm^−1^ (G band), respectively. The G peak corresponds to the sp^2^ hybridization of the carbon network and is attributed to the first order scattering from the E_2g_ phonon modes in the Brillouin zone. Moreover, it originates from the stretching of the sp^2^ carbon pairs in both the rings and chains [30]. The D peak corresponds to the breathing mode of the aromatic rings (disorder-induced modes existing because of structural defects). Therefore, the intensity of the D peak is used to measure the degree of disorder. The positions of the D and G bands were similar for both the GO samples. The L_a_ (defined as the cluster diameter [31] and crystallite size [32]) values of the GO-lf and GO-sf samples were 19 nm and 21 nm, respectively; this difference was not significant. The reduction of GO led to the decrease in the crystallite size, and if a stronger reducer was used, there was a further reduction in the L_a_ value: for the rGO-lr sample (prepared using ascorbic acid), the L_a_ value was 17 nm, and for rGO-hr (prepared using sodium hypophosphite), it was 14.7 nm. The ratio of the intensities of the D and G bands (I_D_/I_G_) determines the reduction degree of GO and provides information about the changes in the sp^2^ and sp^3^ hybridization domains in the carbon lattice [33]. The I_D_/I_G_ values for the GO-sf, GO-lf, rGO-lr, and rGO-hr samples were 0.94, 1.03, 1.16, and 1.33, respectively. This parameter increases with the increase in the degree of disorder, and the increase in the I_D_/I_G_ value is due to the restoration of the sp^2^ hybridization structure. This can be explained by the fact that a greater number of small sp^2^ domains are created after the reduction process. Another D^**^ mode (between D and G) suggests the presence of isolated epoxy groups that form the sp^3^ hybridization in the GO and rGO lattices and can be associated with the phonon state densities (edge effects) or C–H vibrations [34]. The effective reduction of GO is also proved by changes registered in the full width half maximum (FWHM) values, which strongly decreased and caused the D and G peaks to be thin and sharp, suggesting the removal of oxygen groups. Additionally, the bands marked as 2D, D + G, and G + D’ represent structural defects and are disorder activated; additionally, they are connected with phonon scattering and combination around the K point of the Brillouin zone.

#### 2.1.3. XPS Analysis

Partial removal of the oxygen groups and the restoration of sp^2^ hybridization after the reduction process (and therefore the restoration of electrical conductivity) has been proved through the measurement of the resistivity of the samples. A more effective reduction process was performed to obtain the rGO-hr samples, yielding a resistivity value of 0.04 Ω·cm; the mild reduction process (rGO-lr samples) yielded a resistivity value of 0.85 Ω·cm.

**Figure 3:**
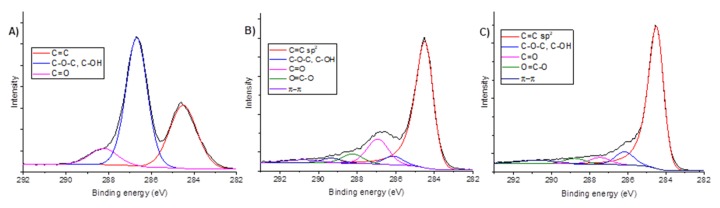
The C1s XPS spectra of the GO-lf (**A**), rGO-lr (**B**), and rGO-hr (**C**) samples. The deconvoluted spectra demonstrate changes in the amount of adequate oxide groups of GO after reduction (**B,C**). Legend: GO-lf: graphene oxide with large flakes (10–20 µm), rGO-lr: reduced graphene oxide with low reduction level, and rGO-hr: reduced graphene oxide with high reduction level.

XPS (Figure 3) was used to investigate the chemical structure of GO and its characteristics after mild (rGO-lr) and strong reduction (rGO-hr). Deconvolution of the C1s peak of the GO-lf samples revealed that its functional oxygen-containing groups mainly consisted of the C–O–C, C–OH (hydroxyl and epoxy at 286.7 eV), and C=O (carbonyl, at 288.2 eV) groups. These peaks decreased after the reduction process and the C=C band that originates from the carbon atoms with sp^2^ hybridization became dominant, indicating that some of the oxygen-containing functional groups are removed and the structure of the double C=C bonds is partially restored. Changes in the C1s/O1s (atomic ratios) values, as calculated from the XPS spectra, were 1.77, 4.75, and 8.50 for the GO, rGO-lr, and rGO-hr samples, respectively; this proves that the highest reduction efficiency was observed in case of the GO samples reduced using sodium hypophosphite. The reduction degree appears to be almost two times higher for the rGO-hr samples than for the rGO-lr samples (Table 1). Because there was no significant difference between the chemical structures of the GO-lf and GO-sf samples (according to the previous observations, e.g., those of the Raman spectroscopy analysis) and because the GO-lf samples were used as the starting materials for the preparation of the rGO-lr and rGO-hr samples, we have included the XPS spectra for the GO-lf samples only.

The results of the XPS analysis are consistent with those of the Raman spectroscopy analysis and electrical measurements, indicating the different oxidation states of the materials. 

### 2.2. GO and rGO May Influence the Morphology of hUC-MSCs 

**Figure 4:**
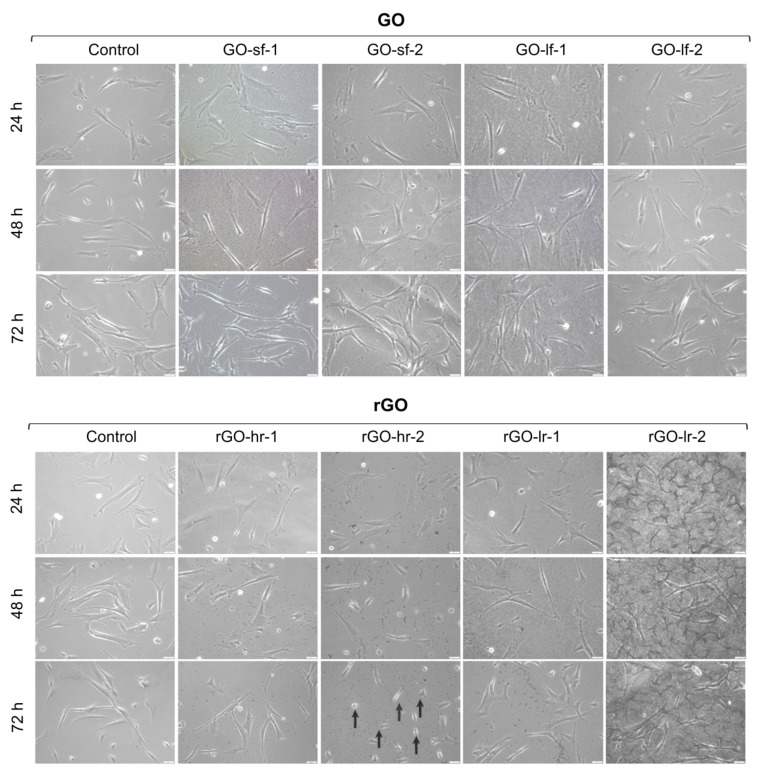
Morphology of the human Wharton’s jelly umbilical cord-derived mesenchymal stem/stromal cells (hUC-MSCs) cultured on the GO and rGO surfaces after 24, 48, and 72 h of culture. Representative images are presented; magnification: 100×. The arrows indicate morphological changes of the hUC-MSCs. Legend: GO-sf-1: small flakes/thick layer; GO-sf-2: small flakes/thin layer; GO-lf-1: large flakes/thick layer; GO-lf-2: large flakes/thin layer; rGO-hr-1: high reduction level/thin layer; rGO-hr-2: high reduction level/thick layer; rGO-lr-1: low reduction level/thin layer; rGO-lr-2: low reduction level/thick layer; Control: tissue culture plastic surface (TCPS).

The modification of cell morphology is one of the most important indicators of the physiological state of cells. Therefore, analysis of the impact of the GO and rGO surfaces on the morphological features of hUC-MSCs was performed. Representative images after 24 h, 48 h, and 72 h of hUC-MSC culture are presented in Figure 4. Our results indicated that all the analyzed surfaces constitute suitable substrates for hUC-MSC propagation. We observed that the GO scaffolds did not alter the morphology of the hUC-MSCs, regardless of the size of the GO flakes, as well as the thickness of the GO layer. All cells exhibited characteristics typical of the morphology of MSCs. Interestingly, notable differences in the shape of the cells cultured for 72 h on a thick layer of highly reduced GO (rGO-hr-2) were observed. Then, the hUC-MSCs exhibited small and oval morphology, compared to the elongated, fibroblast-like cells cultured on a tissue culture plastic surface (TCPS, control sample). These changes suggested the induction of apoptosis and necrosis processes.

### 2.3. The Influence of the GO and rGO Samples on hUC-MSC Proliferation

**Figure 5:**
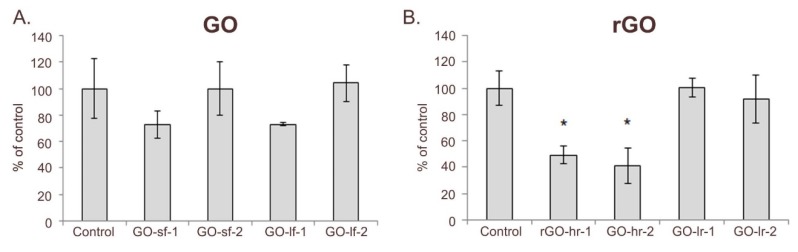
Proliferation of hUC-MSCs after 72 h of cell culture on GO (**A**) and rGO (**B**) substrates. Legend: GO-sf-1: small flakes/thick layer; GO-sf-2: small flakes/thin layer; GO-lf-1: large flakes/thick layer; GO-lf-2: large flakes/thin layer; rGO-hr-1: high reduction level/thin layer; rGO-hr-2: high reduction level/thick layer; rGO-lr-1: low reduction level/thin layer; rGO-lr-2: low reduction level/thick layer; Control: tissue culture plastic surface (TCPS). *P* values less than 0.05 (*p* < 0.05) were considered statistically significant and labeled by an asterisk (*).

Proliferative responses represent a prognostic value of the reaction of cells to a culture condition. Our results evidenced the impact of the GO and rGO samples on the proliferative activity of hUC-MSCs. The results indicated that a thick layer of GO was less favorable for hUC-MSC growth, while the size of the GO flakes did not significantly impact their proliferation (Figure 5A). On the other hand, the number of hUC-MSCs cultured on the slightly reduced rGO surfaces (rGO-lr-1 and rGO-lr-2 samples) was similar to that in case of the control conditions. Interestingly, we observed a significant decrease in the proliferation activity when the cells were cultured on highly reduced rGO scaffolds (Figure 5B). In particular, we observed a decrease in the proliferation rate of hUC-MSCs cultured on: (i) a thin layer (rGO-hr-1) and (ii) a thick layer (rGO-hr-2) of highly reduced GO by 50% and 60%, respectively.

### 2.4. The Influence of the GO and rGO Samples on the Viability of the hUC-MSCs

**Figure 6:**
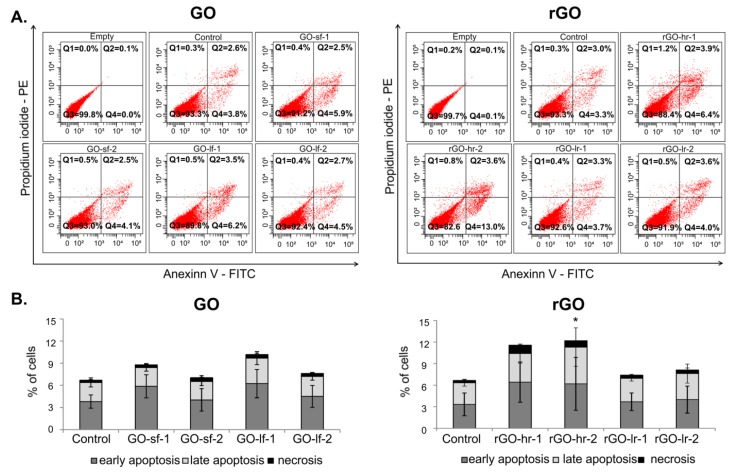
Viability of the hUC-MSCs after 72 h of culture on the GO and rGO substrates. The quantification of cell viability was determined by the flow cytometric analysis of the apoptotic and necrotic cells via the double-staining of hUC-MSCs with Annexin V-FITC and propidium iodide. (**A**) Representative flow cytometric dot-plots are presented to demonstrate the morphology of hUC-MSCs and gating strategy for the determination of the percentages of live (Annexin V-negative and propidium iodide-negative; Q3), early apoptotic (Annexin V-positive and propidium iodide-negative; Q4), late apoptotic (Annexin V-positive and propidium iodide-positive; Q2), and necrotic (Annexin V-negative and propidium iodide-positive; Q1) cells. An unstained probe (empty) constituted the negative control. (**B**) The percentages of early apoptotic, late apoptotic, and necrotic cells were determined using the FACS Diva software. *p* Value less than 0.05 (*p* < 0.05) was considered statistically significant and labeled by an asterisk (*). Legend: GO-sf-1: small flakes/thick layer; GO-sf-2: small flakes/thin layer; GO-lf-1: large flakes/thick layer; GO-lf-2: large flakes/thin layer; rGO-hr-1: high reduction level/thin layer; rGO-hr-2: high reduction level/thick layer; rGO-lr-1: low reduction level/thin layer; rGO-lr-2: low reduction level/thick layer; Control: tissue culture plastic surface (TCPS).

After 72 h of culture on the GO and rGO scaffolds, the analysis of hUC-MSC viability was performed (Figure 6). The obtained results indicated that thin layer of the GO scaffolds (GO-sf-2 and GO-lf-2) had no impact on the cell viability. We observed that the levels of apoptosis in hUC-MSCs cultured on the thin layer of GO (GO-sf-2 and GO-lf-2) were similar to those in the case of the cells cultured on the TCPS (control). On the other hand, thick layer of the GO scaffolds (GO-sf-1 and GO-lf-1) slightly stimulated cell apoptosis. Interestingly, this effect was independent of the size of the GO flakes. We observed about a 30% and 50% increase in the percentage of apoptotic cells when they were cultured on the GO-sf-1 and GO-lf-1 samples, respectively. Moreover, our observation demonstrated that in all tested conditions, the level of necrosis was low, i.e., approximately 0.4% (Figure 6A). 

Furthermore, the analysis of the rGO surfaces revealed that the slightly reduced GO samples did not influence the viability of the hUC-MSCs. Cells cultured on: (i) a thick layer (rGO-lr-1) and (ii) a thin layer (rGO-lr-2) of slightly reduced GO demonstrated similar apoptosis and necrosis levels compared to the cells cultured on the TCPS (control conditions). At the same time, we observed that the hUC-MSCs cultured on: (i) a thin layer (rGO-hr-1) and (ii) a thick layer (rGO-hr-2) of highly reduced GO showed a decreased viability; we observed about 90% more apoptotic cells (in particular, in the early phase of apoptosis), compared to the control conditions (Figure 6B). 

### 2.5. The GO and rGO Samples Maintained the Phenotype of the hUC-MSCs 

**Figure 7:**
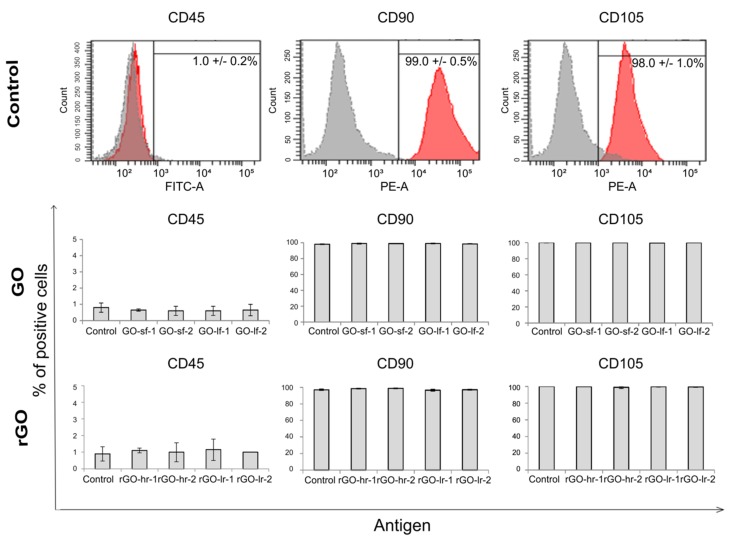
Phenotype characteristics of hUC-MSCs after 72 h of culture on the GO and rGO substrates. The hUC-MSCs were stained with specific monoclonal antibodies conjugated with fluorochromes (CD45-FITC, CD90-PE, CD105-PE) and analyzed using the LSR Fortessa flow cytometer. The representative histograms (for TCPS, panel A), as well as the charts indicating the expression level of the selected antigen (for the GO and rGO surfaces, panel B), are presented. Legend: GO-sf-1: small flakes/thick layer; GO-sf-2: small flakes/thin layer; GO-lf-1: large flakes/thick layer; GO-lf-2: large flakes/thin layer; rGO-hr-1: high reduction level/thin layer; rGO-hr-2: high reduction level/thick layer; rGO-lr-1: low reduction level/thin layer; rGO-lr-2: low reduction level/thick layer; Control: tissue culture plastic surface (TCPS).

To evaluate whether the GO and rGO surfaces impact the expression of the antigens characteristic of mesenchymal cells, flow cytometric analysis was performed. After 72 h of the cell culture on the GO and rGO substrates, the hUC-MSCs were stained with fluorescence-conjugated antibodies against selected antigens that are indicated by the International Society for Cellular Therapy regulations as a typical for mesenchymal stem cells markers: i) positive markers (CD90 and CD105) and ii) negative marker (CD45, antigen characteristic for hematopoietic cells). The results demonstrated that in case of all the studied conditions, hUC-MSCs exhibited a low expression of CD45 (about 1%) and high expression of the CD90 and CD105 antigens (about 98%). Similar results were observed for the cells cultured on the TCPS (control). These data suggest that the GO and rGO scaffolds may lead to the maintenance of the appropriate phenotype of hUC-MSCs regardless of the size of the GO flakes and the level of reduction of the rGO samples (Figure 7).

## 3. Discussion

Nowadays, graphene has emerged as a promising candidate not only useful in the technical field of research, but also for biomedical applications as a substrate for stem cell growth [35]. As previously described, the physicochemical properties of the nanostructured materials may influence the growth, propagation, and viability of cells [36,37]. This effect has also been observed in our experimental model involving graphene-based materials and hUC-MSCs. It is well known that interactions between cells and the culture surfaces are important in many biological processes, including cell adhesion and proliferation [28,38,39]. In particular, in contrast to hydrophobic materials, hydrophilic substrates support cell adhesion and propagation [40,41]; this was also observed in our study. The analysis of several biological aspects indicated that for the growth of hUC-MSCs, hydrophilic GO-based surfaces are more suitable than hydrophobic rGO scaffolds. The obtained results revealed that hUC-MSCs cultured on scaffolds comprising large and small flakes of GO, as well as slightly reduced GO (rGO-lr) scaffolds, demonstrated similar proliferation rates and viabilities as those cultured on TCPS (controls). Moreover, we observed that hUC-MSCs cultured on highly reduced GO surfaces (rGO-hr) showed decreased proliferation compared to those cultured on more hydrophilic GO substrates and TCPS (control). Similar results have been observed by Jin et al., who demonstrated that the viability of hMSCs decreases with the increase in the concentration of rGO [35]. The cytotoxicity impact of highly reduced rGO (rGO-hr) on hUC-MSCs, apart from the mentioned hydrophobicity, can also be explained by the easy formation of reactive oxygen species (ROS) in the presence of rGO-hr. The energy of ROS formation decreases when the oxidation level of GO is lower and when the size of undisturbed graphene network domains increases [42]. Moreover, flakes of rGO-hr possess “sharp” edges, which can cause the cell membrane damage, the loss of membrane integrity and the lipid extraction. The generation of ROS induces oxidative stress and may lead to the damage of cellular proteins and lipids and enhance DNA methylation. As a consequence, the induction of cell apoptosis and necrosis as well as the reduction of the cell viability and proliferation may be detected.

Interestingly, the biofunctionalization of graphene derivatives, as well as the utilization of GO as an additive to biomaterials, may promote cell propagation and enhance the biological activity and differentiation potential of cells. In particular, the incorporation of GO into PLGA (poly (lactic-co-glycolic acid); PLGA) nanofiber scaffolds has been shown to enhance the hydrophilic performance of PLGA and improve the adhesion, proliferation, and osteogenic differentiation of hMSCs [43]. Moreover, the analysis of the morphology of the GO and rGO scaffolds indicated that the rGO samples showed a more agglomerated structure than the GO samples. This porous structure may also be associated with the hydrophobic character of rGO and may influence the behavior of hUC-MSCs. Additionally, the irregular microtopography of culture surfaces, which is observed in the case of graphene-based materials, does not constitute a physiological environment for cells, and may modulate their proliferation, apoptosis, or differentiation [28,44,45]. Thus, detailed studies on GO- and rGO-based nanomaterials as potential scaffolds for stem cell culture are crucial to select the most appropriate material for improving the therapeutic efficiency of hUC-MSCs. The obtained results revealed that unique materials such as graphene are relevant for stem cell propagation. These observations provide a new perspective for research on graphene-based nanomaterials. However, further studies are required to improve their potential utilization in the field of regenerative medicine. 

## 4. Materials and Methods 

Materials. For the experiments, the following chemical compounds were used: graphite (Asbury Graphite Mills Inc., Asbury, NJ, USA) Carbons; with a particle diameter of 300–425 µm), sulfuric acid (POCH S.A., Gliwice, Poland; 96-98%, pure p.a.), orthophosphoric acid (Chempur, Piekary Śląskie, Poland; pure p.a.), potassium permanganate (Chempur, pure p.a.), L(+)-ascorbic acid (POCH S.A., Gliwice, Poland; pure p.a.), sodium hypophosphite monohydrate (Chempur, pure p.a.), hydrochloric acid (Chempur, pure p.a.), perhydrol (Chempur, pure p.a.), and ethanol (POCH S.A., Gliwice, Poland; 96%, pure p.a.). 

### 4.1. GO Preparation

GO was prepared by a modified Hummers method. Graphite flakes were gradually added to a reactor containing concentrated sulfuric acid (H_2_SO4) and orthophosphoric acid (H_3_PO4). Next, potassium permanganate (KMnO4) was slowly added to this mixture; the amount of KMnO4 added was greater than the amount of graphite present in the reactor. The oxidation process was performed for a few hours and stopped by the addition of deionized water, and finally, perhydrol (30% H_2_O_2_). The obtained GO water suspension was left for sedimentation. The purification process was performed using a special installation for microfiltration. Due to specific shearing forces acting on the GO flakes during the purification process, an exfoliation process, which leads to GO formation, occurs. The diameter of the obtained GO flakes varied from 10 to 20 µm and they were named as large flakes (GO-lf). To improve the removal of residual ions, centrifugation and dialysis were performed. To obtain smaller GO flakes, the water GO suspension was subjected to a sonication process. An ultrasonic probe (Sonics&Materials Inc., Newtown, CT, USA) 130 W, amplitude: 40%, probe diameter: 6 mm, 1sec “on”/1sec “off” mode) was used for 5 min; this resulted in the formation of GO flakes with an average size of 0.2–2 µm, and named as small flakes (GO-sf).

### 4.2. rGO Preparation

rGO with a low reduction level (rGO-lr) was prepared via a “green” reduction process by using L(+)-ascorbic acid (C_6_H_8_O_6_) as a reducing agent. The prepared GO-lf layers were placed on polystyrene cell culture plates (6-well plates) and subjected to reduction. First, 2.5 mL of ascorbic acid solution (with a mass concentration of 0.01 g/mL) prepared using water was poured into each well. Reduction was performed for 3.5 h at a temperature of 90 °C on a hot plate. Next, the reducing solution was removed and the obtained rGO-lr layers were rinsed twice with deionized water. 

Highly reduced rGO (rGO-hr) was obtained by using sodium hypophosphite (NaH_2_PO_2_) as a chemical reducer. A solution of sodium hypophosphite (prepared in water) was further acidified using hydrochloric acid; the molar ratio of GO-lf to NaH_2_PO_2_ was 2:1. Reduction was performed for 2 h at a temperature of 95 °C with simultaneous stirring. For this process, aqueous GO suspension was used, and contrary to the case for rGO-lr, GO was reduced in bulk; this was due to the destruction of the GO layers in such conditions. The obtained rGO-hr was rinsed with deionized water to remove residual ions. 

### 4.3. Formation of the GO and rGO Layers

To obtain the most optimal surface for hUC-MSCs growth, GO flakes of different sizes (small and large flakes), GO and rGO suspensions of different thicknesses (thick and thin layers with 10–30 µg/cm^2^ and 3–10 µg/cm^2^, respectively), and GO with different reduction levels (low and high reduction level) were considered. All tested conditions are presented in Table 2.

Based on our previous experience, the use of ethanolic suspensions of GO and rGO leads to a more uniform coverage of the culture plates compared to the water suspensions. To obtain GO layers on six-well plates, the desired amount of GO with a known mass concentration was pipetted into the plates and dried at room temperature, with protection against air fluctuations. For this purpose, water from the aqueous GO suspension was further removed by evaporation at a temperature of 40 °C with simultaneous stirring until a dense paste was formed. Next, ethanol was added and stirred with the GO paste to obtain a homogenous ethanol GO suspension. 

For the fabrication of rGO-lr layers on culture plates, the wells containing the GO layers were reduced, following a previously described procedure. rGO-hr layers were formed by pouring the desired volume of rGO-hr (suspended in ethanol) with a defined mass concentration into the plates. Prior to this, the water suspensions of rGO-hr were filtered on a membrane and pure ethanol was added to replace the water. These suspensions were gently sonicated to obtain homogenous graphene layers. 

### 4.4. Methods for Characterizing GO and rGO

For the investigation of the morphology of the samples, scanning electron microscopy (SEM) was used (Auriga CrossBeam Workstation, Carl Zeiss, Oberkochen, Germany). To examine the chemical structure of GO and rGO, X-ray photoelectron spectroscopy (XPS) was used (UHV Multichamber XPS, Prevac, Rogów, Poland). Analyses of the reduction efficiency and structural changes of the GO and rGO samples were performed using a Raman spectroscopy system (Renishaw Invia, Wotton-under-Edge, UK) at room temperature; the laser was excited at a wavelength of 532 nm and used at a power lower than 1 mW. The electronic properties of GO and rGO were measured using the Hallotron ECOPIA HMS 5500 system.

For SEM and Raman spectroscopy analysis, the graphene materials were deposited onto a Si substrate. For XPS measurements, powdered (freeze-dried) samples were used. For the electrical measurements, freeze-dried samples were pressed into a tablet form.

### 4.5. Isolation and Culture of hUC-MSCs

hUC-MSCs were isolated from human umbilical cord samples provided by The Polish Stem Cell Bank S.A., with permissions from the Polish Ministry of Health (MZ-PZ-TSZ-025-15906-36/AB/14; 11 June 2014). The umbilical cord was washed with sterile PBS (GE Healthcare Life Sciences HyClone Laboratories, South Logan, UT, USA) to remove the blood. Next, the vein and arteries were dissected, and the tissue was cut into small pieces and placed on tissue culture dishes containing DMEM/F12 medium (Sigma-Aldrich, St. Louis, MO, USA) supplemented with 10% FBS (Sigma Aldrich), 100 IU/mL penicillin, and 10 µg/mL streptomycin (Thermo Fisher Scientific, Waltham, MA, USA). After five days, the tissue pieces were removed; the adherent cells were washed with PBS and cultured in complete medium. The cells were cultured in an incubator at 37 °C under conditions of 5% CO_2_ and 95% humidity.

### 4.6. Analysis of hUC-MSCs Morphology

The morphology of the hUC-MSCs cultured on the GO and rGO scaffolds was evaluated using a phase contrast microscope (Olympus IX81; Olympus, Tokyo, Japan) after 24, 48, and 72 h of cell culture. Images were captured using a MicroPublisher 3.3 RTV camera (Teledyne Qimaging, Surrey, BC, Canada) and the Olympus Cell Sens Standard software (Olympus). 

### 4.7. Proliferation Test

The proliferation of the hUC-MSCs cultured on the GO and rGO surfaces was estimated by performing a cell proliferation test. The cells were seeded into six-well plates that were coated with the GO or rGO substrates and contained DMEM/F12 medium (Sigma Aldrich) supplemented with 10% FBS (Sigma Aldrich) at a density of 55 × 10^3^ cells/well. After 72 h of culture, the hUC-MSCs were trypsinized, centrifuged (200 g, 5 min), and counted using a hemocytometer/ Bürker chamber (Paul Marienfeld GmbH & Co.KG, Lauda-Königshofen, Germany). Tissue culture plastic surfaces (TCPS) were used as controls.

### 4.8. Viability Assay

The viabilities of the hUC-MSCs cultured on the GO and rGO substrates were evaluated using a commercially available FITC Annexin V Apoptosis Detection Kit (BD Biosciences, San Jose, CA, USA). First, the hUC-MSCs were seeded into six-well plates that were coated with the GO or rGO samples and contained complete DMEM/F12 medium supplemented with 10% FBS (Sigma Aldrich) at a density of 55 × 10^3^ cells/well. TCPS was used as the control. After 72 h of culture, the cells were harvested and stained with annexin V and propidium iodide according to the manufacturer’s protocol. Cell viability was measured using the LSR Fortessa flow cytometer (BD Biosciences) and the BD FACSDiva 8 software (BD Biosciences).

### 4.9. Phenotype Assessment

To investigate the effects of the GO and rGO scaffolds on the phenotype of the hUC-MSCs, flow cytometric analysis was performed. The hUC-MSCs were seeded into six-well plates that were coated with the GO or rGO substrates and contained complete DMEM/F12 medium supplemented with 10% FBS (Sigma Aldrich) at a density of 55 × 10^3^ cells/well. After 72 h of culture, the hUC-MSCs were harvested, counted, and stained with the following specific monoclonal antibodies conjugated with fluorochromes: CD45-FITC, CD90-PE, and CD105-PE (all procured from Biolegend, San Diego, CA, USA), according to the manufacturer’s protocols. The analysis was performed using the LSR Fortessa flow cytometer (BD Biosciences) and the BD FACSDiva 8 software (BD Biosciences).

### 4.10. Statistical Analysis

All biological experiments were repeated thrice independently. The results are presented as the mean values ± standard deviations (SD). Statistical analysis was performed by one-way ANOVA and Bonferroni test (post hoc test) using the GraphPad Prism software. P values less than 0.05 (*p* < 0.05) were considered statistically significant and labeled by an asterisk (*).

## 5. Conclusions

In this interdisciplinary study, we designed and developed graphene oxide (GO) and reduced graphene oxide (rGO) surfaces dedicated to the culture and propagation of hUC-MSCs. Prior to the in vitro experiments, multiple techniques were used to characterize the obtained GO-based materials. Different sizes of the GO flakes (small and large flakes) and different reduction levels of GO (high and low reduction levels) were examined. Our results indicated that the GO- and rGO-based substrates represented suitable surfaces for hUC-MSC growth and led to the maintenance of their typical mesenchymal morphology and phenotype. Moreover, we observed that the proliferation and viability of hUC-MSCs cultured on GO and rGO surfaces with a low reduction level were similar to those of the cells cultured under the control conditions (tissue culture plate surface, TCPS). However, highly reduced GO scaffolds inhibited the proliferation and decreased the viability of hUC-MSCs; this may be related to the hydrophobic properties of these samples. Thus, the results of our study indicate that GO and rGO with a low reduction level are appropriate and biocompatible for the culture of hUC-MSCs and may have important implications for ex vivo cell expansion dedicated towards tissue repair. Due to the biocompatibility and high electrical conductivity of graphene, GO/rGO surfaces may be potentially used, e.g., as an outer layer of implants dedicated for cardiological applications. The carboxyl and hydroxyl groups in GO structure can be involved in the formation of covalent bonds with different types of molecules and growth factors. Moreover, the antibacterial particles and drugs can also be introduced and stabilized on GO/rGO scaffolds. All of these approaches dedicated for biofunctionalization of GO/rGO surfaces may improve the propagation and differentiation of MSCs to maximize their potential utilization in regenerative medicine applications.

## Figures and Tables

**Table 1:** The results of the XPS analysis for the GO-lf, rGO-lr, and rGO-hr samples. The total atomic percentage of oxygen as well as the atomic percentage of oxygen in the individual oxygen-containing functional groups from the C1s region are presented. Legend: GO-lf: graphene oxide with large flakes (10–20 µm), rGO-lr: reduced graphene oxide with low reduction level, and rGO-hr: reduced graphene oxide with high reduction level.

Sample	Region	Energy (eV)	Atomic %	Energy (eV)	Atomic %	Bonds
**GO-lf**	C 1s	284.5	61.9	284.5	35.4	C=C
	O 1s	530.5	34.9	286.7	55.6	C-O-C, C-OH, C-N
				288.2	9	C=O
**rGO-lr**	C 1s	284.5	82.1	284.5	66	C=C sp^2^
	O 1s	532.5	17.3	286.1	5.2	C-O-C, C-OH
				286.9	16.8	C=O
				288.2	6	O=C-O-
				289.3	3	carbonate
				290.8	3	π-π*
**rGO-hr**	C 1s	284.5	89.2	284.5	77	C=C sp^2^
	O 1s	532.5	10.5	286.2	9.2	C-O-C, C-OH
				287.4	5.2	C=O
				288.8	4.2	O=C-O-
				290.7	4.4	π-π*

**Table 2:** Types of graphene-based materials that were used as substrates for hUC-MSCs growth.

Graphene-Based Substrate	Abbreviation
GO, small flakes, thick layer, 10–30 µg/cm^2^	GO-sf-1
GO, small flakes, thin layer, 3–10 µg/cm^2^	GO-sf-2
GO, large flakes, thick layer, 10–30 µg/cm^2^	GO-lf-1
GO, large flakes, thin layer, 3–10 µg/cm^2^	GO-lf-2
rGO, high reduction level, thin layer 3–10 µg/cm^2^	rGO-hr-1
rGO, high reduction level, thick layer, 10–30 µg/cm^2^	rGO-hr-2
rGO, low reduction level, thin layer, 3–10 µg/cm^2^	rGO-lr-1
rGO, low reduction level, thick layer, 10–30 µg/cm^2^	rGO-lr-2

## Abbreviations

GO	Graphene oxide
rGO	Reduced graphene oxide
MSCs	Mesenchymal stem/ stromal cells
hUC-MSCs	Human Wharton’s jelly umbilical cord-derived mesenchymal stem/stromal cells
